# Efficacy of transcranial magnetic stimulation in treatment-resistant depression

**DOI:** 10.55730/1300-0144.5441

**Published:** 2022-05-07

**Authors:** Kemal AKPINAR, Nalan KALKAN OĞUZHANOĞLU, Tuğçe TOKER UĞURLU

**Affiliations:** 1 Department of Psychiatry, Burdur State Hospital, Burdur, Turkey; 2 Department of Psychiatry, Faculty of Medicine, Pamukkale University, Denizli, Turkey

**Keywords:** Depression, resistance, treatment, transcranial magnetic stimulation

## Abstract

**Background/aim:**

The use of Transcranial Magnetic Stimulation (TMS) in the add-on treatment of patients with treatment-resistant depression (TRD) is becoming more common. This study aims to investigate the efficacy of TMS on depression and accompanying anxiety symptoms among patients with TRD.

**Materials and methods:**

The current study was conducted with 38 patients diagnosed with TRD. The patients were randomly divided into two groups and received 20 sessions of high-frequency (10Hz) TMS and 20 sessions of sham TMS to the left dorsolateral prefrontal cortex in a double-blind and cross-over fashion without a change in their pharmacotherapy. In the clinical evaluation, Hamilton Depression Rating Scale (HAM-D) and Hamilton Anxiety Rating Scale (HAM-A) were carried out three times in total: before, cross-over phase, and at the end of the treatment.

**Results:**

A statistically significant decrease was found in the HAM-D and HAM-A in the group who were actively stimulated in the cross-over phase of the study. While there was a significant decrease in the HAM-A in the group who received sham stimulation, the decrease in the HAM-D was not statistically significant. Group comparisons revealed a statistically significant decrease in HAM-D in the group who were actively stimulated compared to the group receiving sham stimulation. At the end of the study, 63% of 38 patients responded to treatment, 15% partially responded, and 42% reached remission.

**Conclusion:**

This randomized, double-blind, sham-controlled, cross-over study revealed that TMS is superior to sham-TMS, provides clinically significant improvement when implemented besides pharmacotherapy among patients with treatment-resistant depression, and is beneficial for accompanying anxiety symptoms.

## 1. Introduction

Treatment-resistant depression (TRD) is generally defined as a condition where patients with major depressive disorder (MDD) do not respond to at least two distinct and appropriate antidepressant treatments of adequate doses and duration [1,2]. STAR*D (Sequenced Treatment Alternatives to Relieve Depression) study conducted with 4000 patients uncovered that one-third of the patients with MDD could not reach remission even after four aggressive treatment strategies [3]. Treatment strategies include replacing the current antidepressant with a different one from another group, prescribing potentiator drugs (mood stabilizers, atypical antipsychotics, thyroid hormones, psychostimulants), and trying other options such as Electroconvulsive Therapy (ECT). Transcranial Magnetic Stimulation (TMS) treatment is also considered one of the alternative strategies in TRD [4–7].

Transcranial magnetic stimulation (TMS) is a neuromodulation technique that aims to create a magnetic field strong enough to stimulate neurons in the target area. It is used in many psychiatric and neurological diseases for diagnosis and treatment purposes [8,9]. Although the mechanism of action of TMS in depression is not clear, changes in membrane potential, changes in the release of neuromodulators (dopamine, etc.) and neurotrophic factors (BDNF, etc.), neuroplasticity, neurogenesis, cortical excitability, and neuromodulation are assumed [10]. The United States Food and Drug Administration (FDA) approved TMS treatment in patients with TRD in 2008 [11].

Patients who did not respond to treatment despite using two antidepressants, one from the selective serotonin reuptake inhibitor (SSRI) and the other from the serotonin-norepinephrine reuptake inhibitor (SNRI) for at least eight weeks, and currently using SNRI were defined as treatment-resistant depression and included in the study. We aimed to determine the treatment efficacy of randomized, double-blind and cross-over, high-frequency (10Hz) TMS to the left dorsolateral prefrontal cortex (DLPFC) twice a day for a total of 20 sessions and a total of 20 sessions of sham TMS twice a day and examine the effect on anxiety symptoms accompanying MDD.

The present study was based on the thesis study titled “Efficacy of transcranial magnetic stimulation in treatment-resistant depression”.

## 2. Materials and methods

### 2.1. Participants

The study sample consisted of outpatients without any neurological disease between the ages of 18–65 who were admitted to outpatient clinics of Pamukkale University Faculty of Medicine, Department of Psychiatry between August 2018 and April 2019, and diagnosed with MDD in accordance with the DSM-5 diagnostic criteria. All patients who were deemed unresponsive to treatment despite using at least two antidepressants, one from the SSRI and the other from the SNGI, of adequate doses for at least eight weeks and who met the inclusion criteria were invited to participate in the study. The study excluded patients having psychiatric disorders such as psychotic disorder, bipolar disorder, anxiety disorder, alcohol and substance abuse, dementia, and mental retardation, those who are at high risk of suicide, diagnosed with severe MDD with psychotic features, received ECT treatment in the last six months, previously received TMS treatment, and patients with a pacemaker, intracranial implant, foreign bodies, metals or magnetic implants. During the study period, no changes were made in terms of doses or active substances in the treatment regimen for at least eight weeks. Once they received the necessary information, 42 patients with mild major depression who gave their consent to participate were included in the study. The present study was completed with 38 patients. At the beginning of the study, two patients had to leave the study because of transportation issues when visiting the hospital and two others left stating that they did not benefit from the treatment. Patients using 150–300 mg of venlafaxine per day or 60–120 mg of duloxetine per day for at least eight weeks were divided into two groups in a randomized, double-blind fashion. Venlafaxine dosage was adjusted to 75 mg/day for two patients in one group due to sexual side effects and high blood pressure. According to the double-blind technique, the researchers did not know which group the patients belonged to. Only TMS technician knew groups and TMS application (sham or real). The TMS technician, either, had no knowledge of patient data. One (A) of the two groups first received TMS (20,000 pulses in total) and then sham treatment (20,000 sham pulses in total) while the other group (B) was first administered sham treatment (20,000 sham pulses in total) and then TMS (20,000 pulses in total). A total of 20 sessions of cross-over, high-frequency (10Hz) TMS to the left dorsolateral prefrontal cortex (DLPFC) twice a day and a total of 20 sessions of sham TMS twice a day were maintained in both groups for four weeks.

The current study was carried out in accordance with the Declaration of Helsinki and was approved by the Ethics Committee of Pamukkale University with the decision letter dated 17.01.2018 and numbered 601116787-020/4288.

### 2.2. TMS and sham TMS protocol

In this study, the protocol planned to test the efficacy of TMS plus medication versus the efficacy of sham TMS plus medication. TMS treatment protocol was performed in the TMS unit of Pamukkale University Psychiatric Hospital by a psychiatric nurse who has a certificate of practice for TMS, using the Neuro-MS/D (Neurosoft Ltd., Russia) device with figure-of-eight coils, under appropriate physical conditions. In the first session, the resting motor threshold was determined based on the presence of involuntary contractions in the contralateral fingers with a gradual increase in stimulation of the middle interauricular band from the vertex to 5cm lateral. As a result, 110% of the motor threshold was assigned to be the intensity of treatment administration. The site of treatment administration was based on the “5 cm rule”. In line with this rule, 5 cm anterior on the parasagittal plane from the motor cortex point where the motor threshold was determined was regarded as the administration site. This area corresponds to the left DLPFC. The 5cm method is one of the most commonly used and most practical methods to find the projection of the left DLPFC [12]. After assigning the left DLPFC as the administration site, the coil was placed on the scalp at an angle of 45° to the sagittal band.

We know that sham TMS may be administered with the same coil used in actual stimulation by placing the coil away from the scalp at a 45° angle (90° angle to the sagittal band). In this way, patients experience a sound and sensory effect similar to the actual administration; however, the cortical structures below the area where the coil is placed are not stimulated at all [13,14]. The above-mentioned method was utilized for the sham administration, and the same coil and parameters of the actual application were used; however, unlike the actual procedure, the coil was placed away from the scalp at a 45° angle.

Each TMS session was performed in 25 consecutive sequences at 10Hz frequency, with 40 pulses in each sequence lasting 2.5 s, and with a 20-s gap between the sequences. Each session lasted approximately 9–10 min. TMS is administered only on weekdays as 20 sessions for two weeks, two times a day, and 10 sessions a week. The same parameters apply to sham treatment sessions.

### 2.3. Measurement tools

The sociodemographic data form prepared by the researchers was completed with the patients in face-to-face interviews to question their sociodemographic characteristics. In addition, Hamilton Depression Rating Scale (HAM-D) and Hamilton Anxiety Rating Scale (HAM-A) were carried out three times in total: before the treatment, during the treatment (cross-over phase), and at the end of the treatment.

#### 2.3.1. Hamilton Depression Rating Scale (HAM-D)

It is a 17-item scale developed by Hamilton in 1960 to measure the severity of depression. The validity and reliability of its Turkish version are established [15,16]. It is the most common method used to assess the level of depression, symptom distribution, and changes in severity. The total score of the scale ranges from 0 to 53, and high scores indicate an increase in the severity of depression [17].

#### 2.3.2. Hamilton Anxiety Rating Scale (HAM-A)

The scale was developed by Hamilton in 1959 to determine the level of anxiety and symptom distribution in individuals and to measure the changes in severity. The validity and reliability of its Turkish version are ensured. Based on the total score, 17 points or less are considered mild, 18–24 points moderate, and 25 points and above severe [18].

#### 2.3.3 Determining the response to treatment

A decrease of 50% or more in HAM-D scores compared to the initial scores was defined as a response, a decrease of 25%–50% as a partial response, and a decrease of less than 50% as nonresponse. Patients needed to have a HAM-D score of 7 or below in order to achieve “remission”. A HAM-A score of 7 and below, in which the accompanying anxiety level was assessed, was considered a recovery.

### 2.4. Statistical analysis

The data of the present study were analyzed with the SPSS (Statistical Package for the Social Sciences version 22, Chicago, IL, USA) software. Continuous variables are given as mean ± standard deviation while categorical variables as numbers and percentages. Independent samples t-test was used to compare independent group differences when parametric test assumptions were met. On the other hand, Mann-Whitney U test was utilized when parametric test assumptions were not met. When parametric test assumptions were met, paired samples t-test was used, and when these assumptions were not met, Wilcoxon test was performed in dependent groups. Categorical variables were assessed with the Chi-square test. Statistical significance was set at p-value of <0.05 at a 95% confidence interval in all analyses.

## 3. Results

### 3.1. Sociodemographic and clinical characteristics

A total of 38 patients, 32 (84.2%) females and 6 (15.8%) males, were included in the study. The mean age of the patients was 44.6 ± 11.5 (18–65). Table 1 shows the sociodemographic characteristics of the patients who were divided into groups A (first receiving TMS and then sham-TMS) and B (first receiving sham-TMS and then TMS) based on the cross-over of TMS administration in a randomized, double-blind fashion. The mean ages for groups A and B were 43.7 ± 14.2 (18–65) and 45.6 ± 7.8 (33–62), respectively, which is statistically similar (p = 0.608). While the groups were also statistically similar in terms of gender, marital status, educational level, the people they live with, smoking and/or alcohol use (p > 0.05), the employment rate of group B was statistically significantly lower compared to group A (p = 0.039).

The clinical characteristics of all patients are presented in Table 2. The groups were statistically similar in disease onset age, the number of depressive episodes, duration of the last depressive episode, duration of SNRI use for the last depressive episode, history of additional psychiatric illness, history of inpatient treatment, type of SNRI used by the patients during treatment, use of additional psychiatric medication, history of suicide attempt, history of psychiatric illness in first-degree relatives, and history of physical illness (p > 0.05). The venlafaxine and duloxetine dosages of group A were significantly higher compared to the other group (p = 0.004 and p = 0.0001, respectively). Comorbidity was detected in five patients: OCD in remission in two patients, social phobia in one, somatization disorder in one, and impulse control disorder in one.

The pretreatment HAM-D and HAM-A scale scores of the patient groups are presented in Table 3. The two groups were statistically similar (p > 0.05).

### 3.2. Assessment of TMS

Table 3 shows the changes in scale scores before the study (week 0), during the cross-over stage (week 2), and at the end of the study (week 4). When the two groups were analyzed independently of each other, a statistically significant decrease was found in group A compared to HAM-D, both at the end of TMS and at the end of sham-TMS (p = 0.004 and p = 0.034). While there was no significant difference in group B at the end of sham-TMS compared to HAM-D, a statistically significant decrease was found at the end of TMS (p = 0.018).

For group A, HAM-A total score decreased significantly at the end of TMS while HAM-A psychic subscores decreased significantly at the end of sham-TMS (p = 0.027 and p = 0.027). The decrease in HAM-A somatic subscores was not statistically significant at the end of TMS or sham-TMS. For group B, HAM-A total score decreased significantly at the end of sham-TMS while HAM-A psychic subscores decreased significantly at the end of TMS (p = 0.0001 and p = 0.047). The decreases in HAM-A somatic subscores were not statistically significant at the end of TMS or sham-TMS. Decreases in all scales and subscales were statistically significant in both groups before the study (week 0) and at the end of the study (week 4).

When the two groups were compared with each other, HAM-D and HAM-A scale scores at weeks 0, 2, and 4, were found to be statistically similar (p > 0.05) (Table 4). The analyzes conducted by examining the change in the scale scores (Table 5) pointed out that group A’s HAM-D score decreased by 6.85 ± 3.93 points after TMS during the measurements in the weeks 0–2 while there was a decrease of 4.16 ± 4.84 points in group B after sham-TMS. A significant difference was discovered between the decreased rates of HAM-D scores of the two groups after TMS and sham-TMS (p = 0.032). The groups were statistically similar with the changes in HAM-D scores at weeks 2–4 and 0–4 and HAM-A scores compared to all measurement weeks (p > 0.05).

When all patients were divided into two groups based on the type of drug administered (venlafaxine, duloxetine), no significant difference was found between the first and last measured HAM-D scores (p = 0.552 and p = 0.941) and HAM-A (total, psychic, and somatic) scores of the two groups (p > 0.05).

### 3.3. Evaluation of response to treatment

For group A, 6 (30%) patients responded to treatment after 20 sessions of TMS, and a total of 13 (65%) patients responded to treatment with 20 additional sessions of sham-TMS. For group B, 4 (22.2%) patients responded to treatment at the end of sham-TMS, and 7 (38.9%) other patients responded to treatment during TMS. Therefore, a total of 11 (61.1%) patients responded to treatment in this group.

When all of the patients were evaluated as a whole without making any distinctions between the groups, 24 (63%) patients responded to treatment, 6 (16%) patients partially responded, 8 (21%) patients did not respond to treatment, in addition to that, 16 (42.1%) patients reached remission at the end of the study.

### 3.4. Assessment of side effects

No serious side effects were observed in any patient. The most common side effect is a headache with a rate of 21% (n = 8). In addition, 1 (2.6%) of the patients described tinnitus, and 1 (2.6%) reported drowsiness. There was no need for medical intervention in any of the patients who reported side effects. Moreover, no patient discontinued the study for this reason.

## 4. Discussion

As a result of this study, 63% of 38 treatment-resistant depression patients responded to TMS treatment, 15% partially responded, and 42% reached remission. When the groups were compared with each other, a statistically significant decrease was observed in HAM-D in the TMS group compared to the sham TMS group. Also, HAM-A scores decreased in both groups.

HAM-D scores decreased by 6.85 ± 3.93 points in group A and 4.16 ± 4.84 points in group B as a result of the second-week evaluations of the current study. The decrease in scale scores was significantly higher in group A compared to group B. Similarly, Erbay et al. [19] found statistically significant differences in HAM-D scores before and after rTMS. Also, we showed that the rates of response to treatment were significantly higher in the group receiving TMS compared to those receiving sham-TMS. These results suggest that TMS treatment is superior to sham-TMS, which is in line with the results of meta-analyses [11,20].

Although sham-TMS protocol was performed at a 45° angle in the present study, which minimizes the effects of actual stimulation [13], some studies argue that sham-TMS may create partially active stimulation effects that can bring about the intended clinical improvement. Some studies emphasized that sham-TMS has some antidepressant effects, and this placebo effect is generally observed among patients with mild-to-moderate MDD [21].

HAM-D scores decreased significantly in both groups between the 2nd and 4th weeks of the study. This decrease was similar in both groups: 3.85 ± 4.3 points in group A and 6.38 ± 5.84 points in group B. Although there was no statistical difference, the reason why points are higher in group B may be that group B received active TMS treatment in the last 2 weeks of the study. A partial and complete response to treatment increased from 65% to 85% in group A and from 28% to 72% in group B based on HAM-D. These results suggest that TMS is beneficial in TRD. Kedzior et al. [22] investigated the antidepressant effect of TMS without active maintenance therapy in a meta-analysis of 16 double-blind, randomized, sham-controlled studies in the literature. They found that the antidepressant effect persisted longer in the follow-up of patients who have had lower disease severity, unipolar depression, no psychotic symptoms, showed resistance to treatment, and taken antidepressant medication at the beginning. In long-term follow-up (8–16 weeks), the efficacy was lower compared to those with a short (1–4 weeks) follow-up period [22]. A study conducted by Theleritis et al. revealed a further decrease in HAM-D scores in the 2-week follow-up of 89 patients with TRD after the 3-week TMS treatment. This decrease has been attributed to a delayed effect of TMS in some patients [23].

At the end of the 2nd week, there was a significant decrease in HAM-A scale total scores in both groups while the psychic and somatic subscales declined at a nonsignificant level. When the anxiety level of the patients was compared between the groups, there were similarly minimum decrease rates at the end of the 2nd week. This indicates that TMS was not superior to sham-TMS with regards to anxiety symptoms accompanying depression in the current study. While some studies [24] support this result, there are many others [25,26] that argue the opposite in the literature. According to the results of a recent meta-analysis, TMS is effective in generalized anxiety disorder, but this field needs to be supported by more research [27].

At the end of the current study, both groups experienced a decrease in the initial and final HAM-D and HAM-A scores. Patients (63%) responded to treatment and 42.1% achieved remission. When we look at the literature, similar to our study, it is mentioned that TMS is superior to sham TMS in response and remission rates, with an average of 30%–60% [6,28]. Bolu et al. [28] found that at similar ages, response rates were 26.1%–44.7% and MDD responds to treatment better than other psychiatric disorders.

The majority of the patients in the study were female (n = 32) and middle-aged 44.6 ± 11.5 (18–65) individuals. Epidemiological studies generally emphasize that major depressive disorder is more common, especially, in the middle-aged group, and is twice as common among women 1 Lingeswaran showed that the antidepressant effect of TMS treatment is more prominent at a younger age [29]. It has been reported that the brain undergoes atrophy and the distance between the scalp and the prefrontal cortex increases due to aging, reducing the electrical activity of the magnetic field created by TMS in the targeted cortical tissue, which in turn causes fewer neuroplastic changes [30].

All the patients in our study continued the antidepressant treatment they were receiving before TMS started at the same dose. For group A, the mean daily doses of both venlafaxine and duloxetine were significantly higher compared to the other group. Although this result is due to randomization, it leads to questioning the level of efficacy between the 2 groups. In this study, no differences were observed in the treatment of TMS by medication type. For group B, two patients whose venlafaxine dosage was reduced (75mg/day) due to side effects also responded to TMS treatment. Most of the studies reported that the application of TMS alongside existing pharmacological treatment has a higher treatment efficacy than those which only provide TMS [31,32]. The current study administered TMS in addition to pharmacotherapy to achieve a better response to treatment.

All patients tolerated TMS successfully with the following specifications: 2 sessions per day with a frequency of 10Hz (high), 110% motor threshold, and 1000 pulses per session. Nonserious side effects were observed in 10 of the patients. In the present study, the most common side effect was headache, which is consistent with the literature [11]. Headache may be caused due to having to maintain the posture during the application and the magnetic field. Contrary to studies pointing out that the application of TMS at high frequencies and in short intervals is feasible and safe, some studies report that the risk of seizure, one of the most serious side effects, increases in these conditions [11].

O’Reardon et al. extended the duration of TMS treatment to 4–6 weeks and found that the efficacy of the treatment increased with longer periods of stimulation [33]. However, as the duration of treatment is increased, the rate of discontinuation also increases [34]. The current study implemented a 4-week treatment period to minimize discontinuation rates. It has been suggested that more sessions of TMS and a higher number of pulses per day may accelerate the antidepressant effect and decrease the rates of discontinuation during the TMS treatment [11]. Four of the 42 patients left at the beginning of the present study, and there was no discontinuation in the following period. In their sham-controlled study where 1 and 2 sessions of TMS treatment were administered per day, Theleritis et al. [23] found that patients who received 2 sessions of TMS per day had a higher response and remission rates than those who received TMS once a day after 3 weeks of treatment. The number of sessions and daily pulses are important factors that increase the efficacy of treatment. While evaluating the positive effect of increasing the number of TMS daily sessions on remission rates, there is also information that anxiety and irritability may increase [10].

In studies investigating the efficacy of TMS among patients with TRD are quite heterogeneous. In treatments administering different time and different pulse numbers each have a positive effect on the treatment and remission rates to a varying degree [35–37]. The rates of TMS treatment response varies, which may be caused by many reasons. For instance, as the number of pulses, motor threshold, frequency, and duration of TMS application the lack of standardization regarding the indications and protocol of TMS application is a crucial issue, it is not possible to guarantee that the region to be stimulated (DLPFC) is correctly determined. Another issue is that methods to manage clinical differences or the predictors of response to treatment are not known because the mechanism of action of TMS is not yet clearly defined. Differences in psychotherapy and/or pharmacotherapy received by patients during and after TMS, tolerance to treatment, the approach assumed by healthcare professionals, and the differences in the evaluation scales used in the studies are other important issues to consider. In the present study, patients’ beliefs about a new treatment, spending one-to-one more time with patients during the application, and the high number of patients with moderate MDD may have increased the efficacy of TMS treatment.

Limitations of the current study include the following: the predominance of female patients participating in the study, the small number of samples, and the inability to isolate the effect of TMS due to its administration alongside the drug treatment. Also, the neuro-navigation method and sham coil are not used for determining the area where TMS will be applied because of their high cost. Another limitation is that the follow-up studies of the patients are not part of the current study.

In conclusion, TMS is a low-cost, well-tolerated somatic treatment method with few side effects in TRD where the treatment rate is low with current pharmacotherapies. The present study demonstrated that TMS, in its randomized, double-blind, sham-controlled cross-over method, is superior to sham-TMS and is beneficial for depression and accompanying anxiety symptoms. However, further research is needed in different depression subgroups with larger sample sizes and supported by follow-up studies.

## Figures and Tables

**Table 1:** Sociodemographic characteristics of groups.

		Group A		Group B		p
	Mean±SD		Mean±SD			
Age		43.7 ± 14.2		45.6 ± 7.8		0.608
		n	%	n	%	
**Gender**	**Female**	17	85	15	83.3	0.888
	**Male**	3	15	3	16.7	
**Marital status**	**Single**	5	25	0	0	0.071
	**Married**	10	50	13	72.2	
	**Divorced**	5	25	5	27.8	
**Education**	**Primary school**	12	60	7	38.9	0.574
	**Secondary school**	2	10	2	11.1	
	**High school**	2	10	4	22.2	
	**University**	4	20	5	27.8	
**People living with**	**Alone**	4	20	2	11.1	0.453
	**With family**	16	80	16	88.9	
**Working status**	**Unemployed**	9	45	14	77.8	**0.039**
	**Working**	11	55	4	22.2	
**Smoking**	**No**	16	80	14	77.8	0.867
	**Yes**	4	20	4	22.2	
**Drinking alcohol**	**No**	19	95	17	94.4	0.939
	**Yes**	1	5	1	5.6	

**Table 2:** Clinical characteristics of the groups.

Clinical characteristics		Group A		Group B		p
**Disease onset age (Mean**±**SD)**		33.8 ± 14.4		31.8 ± 8.7		0.849
**Number of depressive episodes (n, %)**		3.1 ± 1.8		2.8 ± 2		0.875
**Last depressive episode duration (month) (Mean**±**SD)**		5.1 ± 3.2		5.3 ± 2.8		0.964
**SNRI usage time in the last episode (week) (Mean**±**SD)**		17.5 ± 14.9		18.8 ± 19.5		0.930
**Psychiatric illness history (n, %)**	**No**	17	94.4	16	80	0.188
	**Yes**	1	5.6	4	20	
**Inpatient history (n, %)**	**No**	17	94.4	17	85	0.344
	**Yes**	1	5.6	3	15	
**Suicide (n, %)**	**No**	14	77.8	17	85	0.566
	**Yes**	4	22.2	3	15	
**Psychiatric illness history (1st-degree relatives) (n, %)**	**No**	11	61.1	10	50	0.540
	**Depression**	5	27.8	5	25	
	**Other psychiatric illness**	2	11.1	5	25	
**Physical illness history (n, %)**	**No**	7	38.9	13	65	0.107
	**Yes**	11	61.1	7	35	
**SNRI (n, %)**	**Venlafaxine**	12	66.7	9	45	0.180
	**Duloxetine**	6	33.3	11	55	
**Venlafaxine dose (Mean** ± **SD)**		225±64.9		150±45.2		**0.004**
**Duloxetine dose (Mean** ± **SD)**		92.7±28.3		80±30.9		**0.0001**
**Psychiatric treatment (n, %)**	**No**	12	66.7	10	50	0.299
	**Yes**	6	33.3	10	50	

**Table 3:** Evaluation of the changing scale scores of the groups during the treatment process.

Group	Process / p	HAM-D	HAM-A		
			Psychic	Somatic	Total
		Mean±SD	Mean±SD	Mean±SD	Mean±SD
**Group A**	**0.week**	20.2 ± 3.3	7.7 ± 3.1	7.8 ± 4.5	15.5 ± 6.9
	**2.week**	13.4 ± 5.7	5.3 ± 2.4	5.5 ± 3.8	11.5 ± 5.5
	**4.week**	9.5 ± 5.1	3.4 ± 2	3.9 ± 3	7.3 ± 4.7
	**p** **_1_**	**0.004** *	0.291	0.707	**0.027** *
	**p** **_2_**	**0.034** *	**0.027** *	0.098	0.291
	**p** **_3_**	**0.0001** *	**0.0001** *	**0.003** *	**0.0001** *
**Group B**	**0.week**	20.5 ± 3.4	7 ± 1.4	6.4±2.5	13.4±2.9
	**2.week**	16.3 ± 6.9	4.7 ± 1.6	4.5±2.7	8.5±3.5
	**4.week**	9.9 ± 7.6	3 ± 1.7	2.8±2.4	5.9±3.8
	**p** **_1_**	0.073	0.166	0.073	**0.0001** *
	**p** **_2_**	**0.018** *	**0.047** *	0.137	0.116
	**p** **_3_**	**0.0001** *	**0.0001** *	**0.0001** *	**0.0001** *

p1: p-value between prestudy–2. weekp2: p-value between 2.week–4.weekp3: p-value between prestudy and 4 weeks

* p < 0.05

**Table 4:** Evaluation of the changing scale scores during the treatment process according to the groups.

Scale		Process	Group A (Mean±SD)	Group B (Mean±SD)	p
**HAM-D**		**0.week**	20.2 ± 3.3	20.5 ± 3.4	0.823
		**2.week**	13.4 ± 5.7	16.3 ± 6.9	0.164
		**4.week**	9.5 ± 5.1	9.9 ± 7.6	0.758
**HAM-A**	**Psychic**	**0.week**	7.7 ± 3.1	7 ± 1.4	0.613
		**2.week**	5.3 ± 2.4	4.7 ± 1.6	0.582
		**4.week**	3.4 ± 2	3 ± 1.7	0.529
	**Somatic**	**0.week**	7.8 ± 4.5	6.4 ± 2.5	0.274
		**2.week**	5.5 ± 3.8	4.5 ± 2.7	0.668
		**4.week**	3.9 ± 3	2.8 ± 2.4	0.282
	**Total**	**0.week**	15.5 ± 6.9	13.4 ± 2.9	0.566
		**2.week**	11.5 ± 5.5	8.5 ± 3.5	0.053
		**4.week**	7.3 ± 4.7	5.9 ± 3.8	0.316

**Table 5:** Evaluation of the difference between the scale scores in the treatment process according to the groups.

Scale		Process	Group A (Mean±SD)	Group B (Mean±SD)	p
**HAM-D**		**0. week–2. Week**	6.85 ± 3.93	4.16 ± 4.84	**0.032** *
		**2. week–4. Week**	3.85 ± 4.3	6.38 ± 5.84	0.171
		**0. week–4. Week**	10.7 ± 5.19	10.55 ± 6.6	0.940
**HAM-A**	**Psychic**	**0. week–2. Week**	2.35 ± 3.18	2.22 ± 2.36	0.890
		**2. week–4. week**	1.9 ± 2.42	1.72 ± 1.7	0.857
		**0. week–4. Week**	4.25 ± 3.69	3.94 ± 2.6	0.772
	**Somatic**	**0. week–2. week**	2.3 ± 3.46	1.94 ± 2.79	0.732
		**2. week–4. week**	1.6 ± 2.28	1.61 ± 1.78	0.784
		**0. week–4. Week**	3.9 ± 4.27	3.55 ± 3.27	0.784
	**Total**	**0. week–2. week**	3.95 ± 4.46	4.94 ± 3.65	0.214
		**2. week–4. week**	4.2 ± 4.69	2.55 ± 4.84	0.295
		**0. week–4. week**	8.15 ± 7.15	7.5 ± 4.86	0.748

* p < 0.05

## Footnotes

1 World Health Organization (2017). Depression and Other Common Mental Disorders Global Health Estimates [online]. Website https://apps.who.int/iris/bitstream/handle/10665/254610/WHO-MSD-MER-2017.2-eng.pdf [accessed 01 June 2022].
